# Senescence of bone marrow-derived mesenchymal stem cells from patients with idiopathic pulmonary fibrosis

**DOI:** 10.1186/s13287-018-0970-6

**Published:** 2018-09-26

**Authors:** Nayra Cárdenes, Diana Álvarez, Jacobo Sellarés, Yating Peng, Catherine Corey, Sophie Wecht, Seyed Mehdi Nouraie, Swaroop Shanker, John Sembrat, Marta Bueno, Sruti Shiva, Ana L. Mora, Mauricio Rojas

**Affiliations:** 1 Dorothy P. & Richard P. Simmons Center for Interstitial Lung Disease, University of Pittsburgh School of Medicine, W1244 BST Tower 200 Lothrop Street, Pittsburgh, PA 15261 USA; 2 Division of Pulmonary, Allergy and Critical Care Medicine, University of Pittsburgh School of Medicine, Pittsburgh, PA USA; 3 Interstitial Lung Disease Program, Hospital Clinic, Barcelona, Spain; 4 Research Unit of Respiratory Diseases, Central South University, Changsha, 410011 Hunan China; 5 Vascular Medicine Institute of the University of Pittsburgh, University of Pittsburgh School of Medicine, Pittsburgh, PA USA; 6 Department of Pharmacology & Chemical Biology, University of Pittsburgh, Pittsburgh, PA USA

**Keywords:** Idiopathic pulmonary fibrosis, Mesenchymal stem cells, Aging, Cellular senescence, And mitochondria

## Abstract

**Background:**

Idiopathic pulmonary fibrosis (IPF) is a chronic lung disease for which age is the most important risk factor. Different mechanisms associated with aging, including stem cell dysfunction, have been described to participate in the pathophysiology of IPF. We observed an extrapulmonary effect associated with IPF: increase in cell senescence of bone marrow-derived mesenchymal stem cells (B-MSCs).

**Methods:**

B-MSCs were obtained from vertebral bodies procured from IPF patients and age-matched normal controls. Cell senescence was determined by cell proliferation and expression of markers of cell senescence p16^INK4A^, p21, and β-galactosidase activity. Mitochondrial function and DNA damage were measured. Paracrine induction of senescence and profibrotic responses were analyzed in vitro using human lung fibroblasts. The reparative capacity of B-MSCs was examined in vivo using the bleomycin-induced lung fibrosis model.

**Results:**

In our study, we demonstrate for the first time that B-MSCs from IPF patients are senescent with significant differences in mitochondrial function, with accumulation of DNA damage resulting in defects in critical cell functions when compared with age-matched controls. Senescent IPF B-MSCs have the capability of paracrine senescence by inducing senescence in normal-aged fibroblasts, suggesting a possible link between senescent B-MSCs and the late onset of the disease. IPF B-MSCs also showed a diminished capacity to migrate and were less effective in preventing fibrotic changes observed in mice after bleomycin-induced injury, increasing illness severity and proinflammatory responses.

**Conclusions:**

We describe extrapulmonary alterations in B-MSCs from IPF patients. The consequences of having senescent B-MSCs are not completely understood, but the decrease in their ability to respond to normal activation and the risk of having a negative impact on the local niche by inducing inflammation and senescence in the neighboring cells suggests a new link between B-MSC and the onset of the disease.

**Electronic supplementary material:**

The online version of this article (10.1186/s13287-018-0970-6) contains supplementary material, which is available to authorized users.

## Background

Idiopathic pulmonary fibrosis (IPF) is a chronic interstitial lung disease characterized by a progressive and irreversible loss of lung function though accumulation of scar tissue [1–3]. Its annual incidence in the USA has been estimated to be 6.8–16.3 cases per 100,000 inhabitants [1, 4]. IPF has a heterogeneous evolution and, even though periods of clinical stability may be observed, progressive deterioration is unavoidable with a median survival of 3–5 years from the time of diagnosis [5]. Although two new approved therapies are currently available (pirfenidone and nintedanib), their efficacy is limited, and several adverse effects have been described [6].

Aging is considered the main risk factor for IPF [7–11]. Along with others, we have demonstrated that there is an increase in markers of cell senescence in lung fibroblasts from IPF patients [12–15]. Additionally, we have shown that, in animal models of lung injury, aged bone marrow-derived mesenchymal stem cells (B-MSCs) have decreased protective activity [16]. This is in contrast to what we had previously described in young animal models of pulmonary fibrosis, where infusion of B-MSCs isolated from normal young donors in the initial stages of the injury results in a decrease in collagen deposition in the lung after bleomycin instillation [17, 18]. Therefore, we aimed to determine the differences in the biological and functional characteristics of B-MSCs from healthy individuals and IPF patients within the same age range. Characterization of IPF B-MSCs shows an increase in cell senescence linked to an upsurge of senescence-associated secretory phenotypes (SASPs) promoting a proinflammatory milieu and increasing deposition of components from the extracellular matrix. Our data suggest that extrapulmonary alterations in B-MSCs from IPF patients might contribute to the pathogenesis of the disease. To our knowledge, this is the first report describing amelioration in functional and reparative capacities of the endogenous nonpulmonary MSCs from patients who have developed IPF.

## Methods

### Human B-MSC isolation and manipulation

Human B-MSC isolation was approved by the Committee for Oversight of Research and Clinical Training Involved Decedents (CORID) of the University of Pittsburgh. As previously described, B-MSCs were isolated from bone marrow fragments from cadaveric vertebral bones [16]. B-MSCs were divided into three groups: young donors (18–30 years; *n* = 7), old donors (57–82 years; *n* = 11), and IPF patients (60–82 years; *n* = 8) (Additional file 1: Table S1). B-MSCs were isolated, cultured, and expanded according to previously published protocols (see Additional file 1: Online data supplement).

### Animals and animal treatment

Female 11-week-old C57BL/6 mice (The Jackson Laboratory, Bar Harbor, ME) were treated with 2 U/kg bleomycin hydrochloride solution (63323–136-10; APP pharmaceuticals, Schaumburg, IL) dissolved to 1 U/ml in sterile saline and delivered by direct injection into the trachea using a 0.9-mm needle. While under anesthesia, a cell suspension of 500,000 human B-MSCs in 100 μL culture medium was injected intravenously. Control group mice received the same volume of sterile medium solution (see Additional file 1: Online data supplement for details). All animal protocols were reviewed and approved by the Institutional Animal Care and Use Committee (IACUC).

### Statistical analysis

Statistical analyses were performed using GraphPad Prism version 7 (GraphPad Software) and STATA version 13 (Stata Corporation). Comparisons between control and IPF B-MSCs were made using a Mann-Whitney test. Kruskal-Wallis and Dunn tests were used for between-group comparisons. For time-dependent observations, we used mixed-effect models with a robust variance estimator to calculate the difference between groups in experimental outcomes.

## Results

### IPF B-MSCs are more senescent than age-matched control B-MSCs

**Fig. 1:**
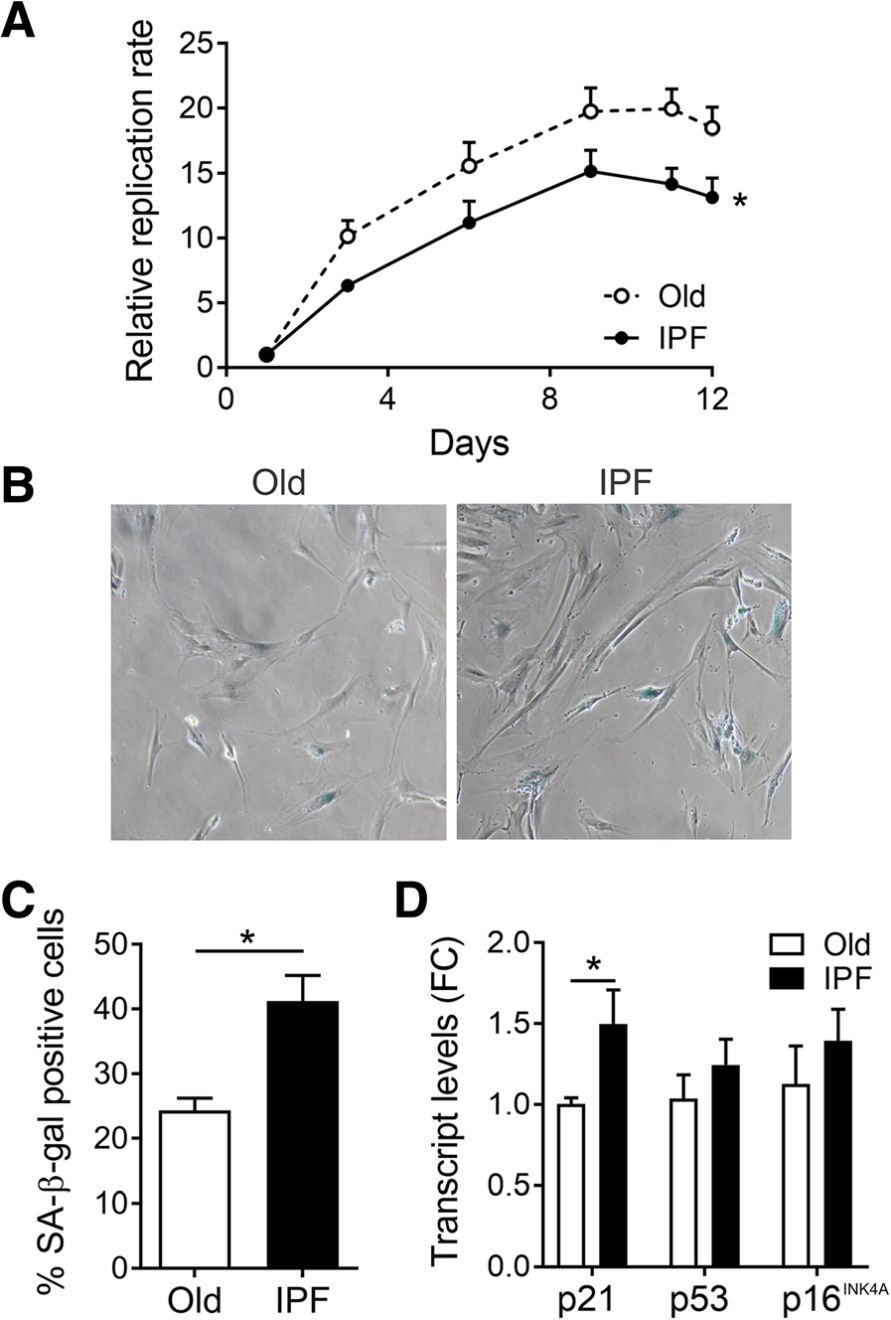
B-MSCs from IPF patients show increased senescence. **a** Proliferation rates of idiopathic pulmonary fibrosis (IPF) B-MSC (filled circles) compared with age-matched controls (Old; open circles), showing slower proliferation rates in IPF B-MSCs compared with age-matched controls. **b** Representative photos of B-MSCs subjected to Senescence-associated β-galactosidase (SA-β-gal) staining. **c** Percentage of SA-β-gal-positive cells from IPF patients and age-matched controls (Old); mean ± SEM; *n* = 3. **d** Expression of senescence markers p21, p16^INK4A^, and p53 measured by qRT-PCR; fold change (FC) ± SEM; *p < 0.05

B-MSCs isolated from IPF patients showed morphological changes characterized by increased cell size accompanied by replicative senescence in comparison with B-MSCs from age-matched controls. Quantification of B-MSC proliferation by measurement of DNA staining showed decreased cell proliferation (Fig. 1a), which was confirmed by determination of flow cytometric quantification of cell cycle phases. A lower number of IPF B-MSCs was observed in the G2/M phase compared with old control B-MSCs and a higher number of IPF-B-MSCs were found in the G0 phase after transforming growth factor (TGF)-β1 stimulation (Additional file 1: Supplementary Figure S1).

To confirm the senescence phenotype of B-MSCs from IPF patients, we investigated the presence of other markers of senescence. Measurements of senescence-associated β­galactosidase (SA-β-gal) activity showed significantly higher positivity in B-MSCs derived from IPF patients compared with the control group (Fig. 1b, c). Furthermore, transcript levels of the inhibitor of cell cycle p21 was significantly increased in IPF B-MSCs and p16^INK4A^ and p53 were moderately increased when compared with age-matched controls [19, 20] (Fig. 1d).

### DNA damage in B-MSCs from IPF patients

**Fig. 2:**
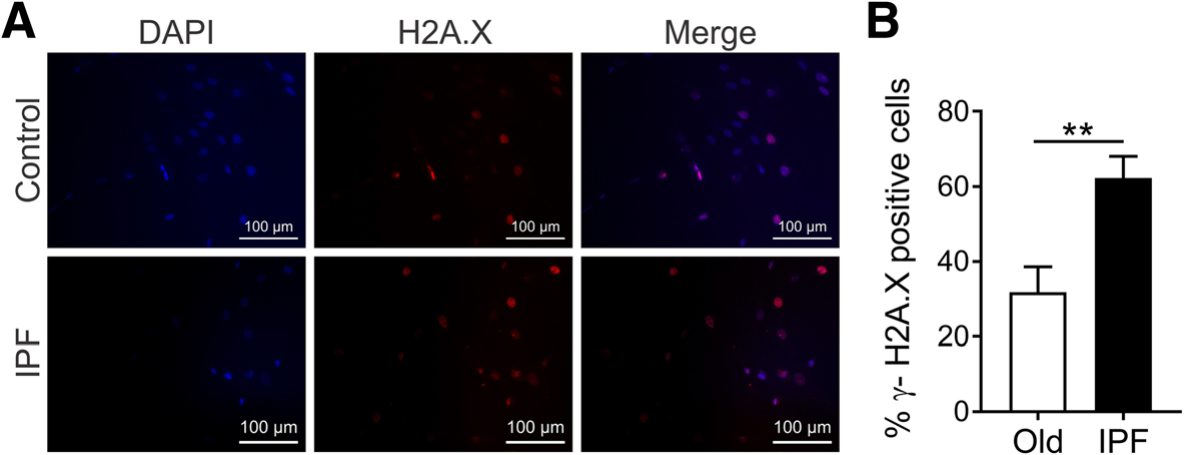
B-MSCs from IPF patients show accumulation of DNA damage. Immunofluorescent analysis of histone H2AX phosphorylation (H2A.X) was performed. **a** Representative images are shown for control (Old) and patient (idiopathic pulmonary fibrosis (IPF)) cells (γH2AX, red; DAPI, blue). **b** Results are expressed as the percentage of the γH2AX-positive cells, showing significantly more γH2AX-positive IPF B-MSCs than old control cells; mean ± SEM; *n* = 10; **p ≤ 0.01

The ability to repair DNA declines with age, and the consequent accumulation of DNA damage leads the cells to senescence or apoptosis [21]. We evaluated DNA damage in B-MSCs from IPF patients and controls by determination of γ-H2AX phosphorylation (Fig. 2a). Quantification of positive cells showed a significantly higher percentage of IPF B-MSCs with DNA damage compared with age-matched controls (Fig. 2b). Additionally, telomere shortening has been identified as one of the hallmarks of aging [21]. We observed an important tendency to a shorter telomere length in B-MSCs from IPF patients when measured by flow-FISH [22] (Additional file 1: Figure S2) that correlates with a senescent phenotype.

### Decreased stemness and function of IPF B-MSCs

**Fig. 3:**
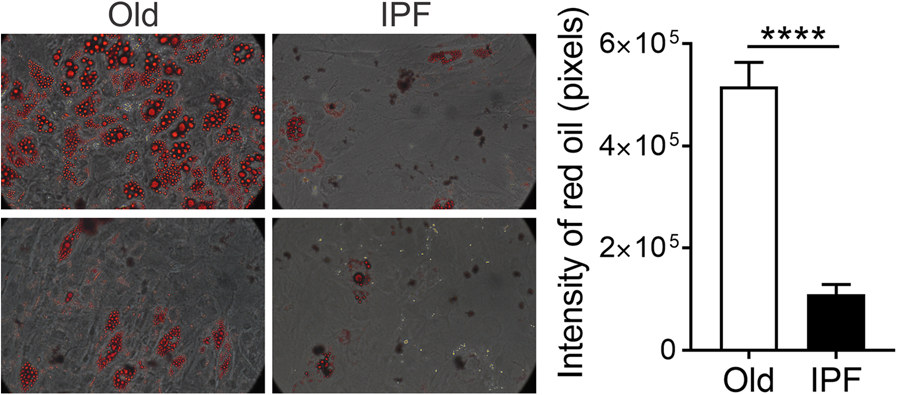
B-MSCs from IPF patients show a decline in their ability to differentiate. In-vitro adipose cell differentiation was induced in B-MSCs from age-matched controls (Old) and idiopathic pulmonary fibrosis (IPF) patients. Cells were incubated with adipose differentiation media for 15 days, fixed, and stained with Oil Red O. Differentiation was assessed quantitatively by microscopic analysis of red pixels to measure lipid accumulation relative to the control samples; mean ± SEM; ****p ≤ 0.0001

B-MSCs are characterized by their ability to differentiate into chondrocytes, osteocytes, and adipocytes [18, 23, 24]. With age, the differentiation potential is attenuated or inhibited for chondrogenesis and osteogenesis. Conversely, the differentiation potential into the adipogenic lineage is increased with senescence [25]. We evaluated the ability of B-MSCs to differentiate by treating them with adipogenic media. After 21 days of treatment, quantification of Oil-Red staining demonstrated a significant decrease in positive cells in cell cultures of IPF B-MSCs compared with the age-matched control, suggesting that the differentiation capacity was diminished in IPF-B-MSCs (Fig. 3)**.**

TGF-β1 also plays an important role in directing fate decision in B-MSCs and modulating regenerative function of B-MSCs [26–28]. We analyzed the response to TGF-β1 stimulation on control and IPF B-MSCs at 24 h and 72 h. At 24 h, IPF-MSCs expressed higher levels of interleukin (IL)-6 (a well-known factor of SASP) than control cells (data not shown). In sharp contrast, at 72 h of TGF-β1 stimulation, transcript levels of growth factors associated with wound healing and reduction of tissue fibrosis such as TGS-6, KGF, and IL-1RN were diminished in IPF B-MSCs (data not shown).

**Fig. 4:**
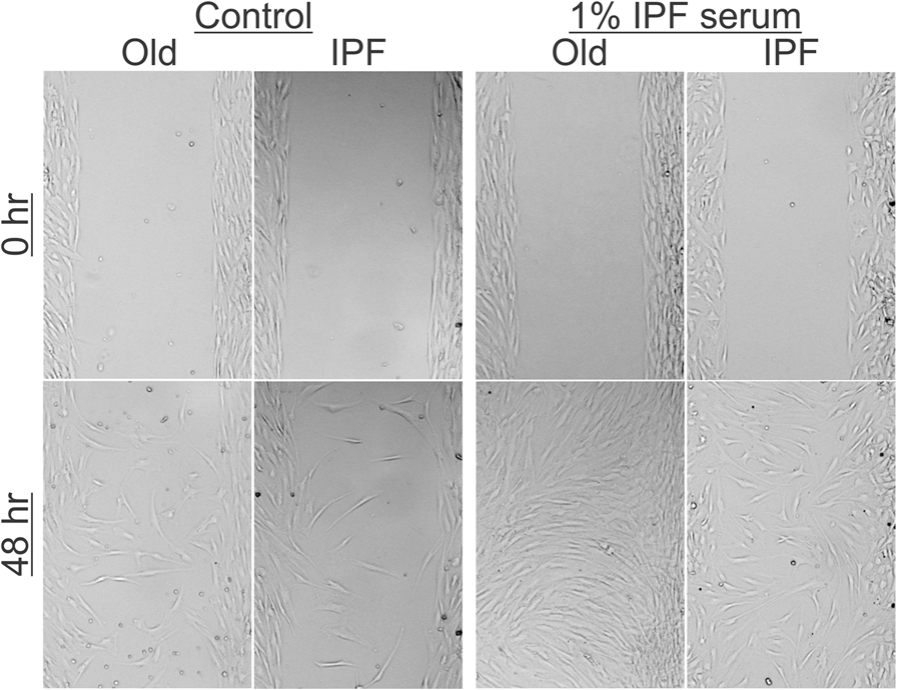
IPF B-MSC migration is impaired. Cell migration was determined by creating a wound gap and the progression of wound closure was photographed using an inverted microscope. Idiopathic pulmonary fibrosis (IPF) and age-matched (Old) B-MSCs were incubated in growth medium with or without 1% IPF serum (*n* = 3) for 48 h

The wound healing process is also affected by the capacity of B-MSCs to migrate to the injured organ [16]. Using a combination of parabiosis and bleomycin-induced lung fibrosis models, we first analyzed the effect of aging for in-vivo migration of B-MSCs (see Additional file 1: Online data supplement). We found that bleomycin-injured lungs, independent of the age, can generate the appropriate signals which promote the recruitment of cells that express a pattern of surface markers that resemble B-MSCs into the lung. However, only B-MSCs from young mice were able to migrate and home into the injured lung, suggesting an age-related defect of B-MSCs to respond to chemotactic stimuli (Additional file 1: Figure S3). Secondly, in-vitro studies were used to analyze the migration capacity of IPF B-MSCs. Migration and proliferation of IPF B-MSCs and controls were determined by in-vitro wound closure assays. Control B-MSCs were able to close the wound after 48 h of stimulation with 1% of serum from IPF patients. On the contrary, IPF B-MSCs failed to close the wound with the same stimuli (Fig. 4). Both control and IPF B-MSCs have minimal migration after 24 h of TGF-β1 stimulation or media without serum (data not shown).

### IPF B-MSCs have fragmented and dysfunctional mitochondria

**Fig. 5:**
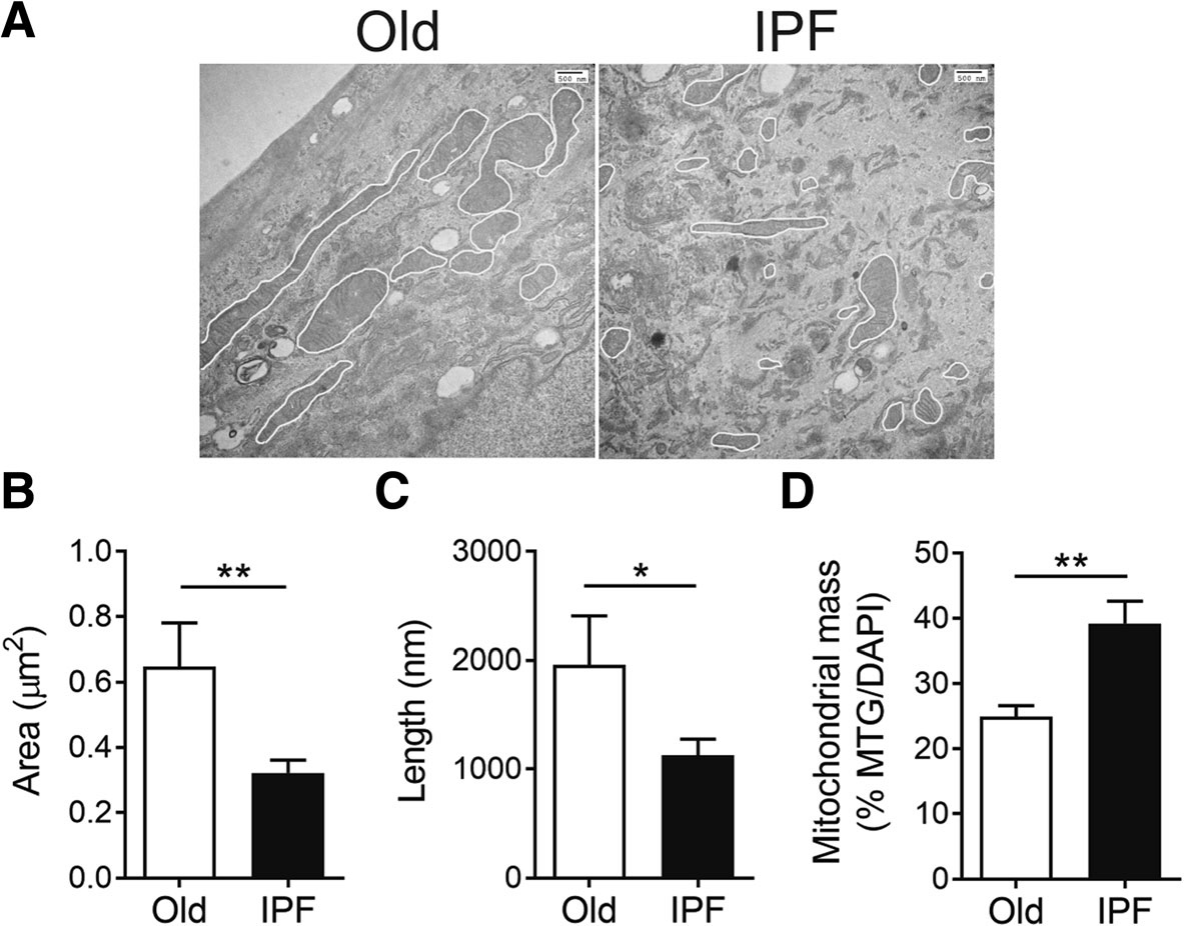
Mitochondria in B-MSCs from IPF patients are more abundant but smaller compared with aged-matched controls. **a** Transmission electron microscopy (TEM; *n* = 2 and 4 per group) of B-MSCs from donor control (Old) and idiopathic pulmonary fibrosis (IPF) patients. Scale bars = 500 nm. **b**,**c** Quantitative analysis of morphometric data from TEM images (area and length). **d** Mitochondrial mass determined by MitoTracker Green (MTG) and normalized to cell number using DAPI; *p ≤ 0.05, **p ≤ 0.01

Mitochondrial dysfunction has been implicated in the induction of cellular senescence and fibrosis [8, 29, 30]. It is also recognized that mitochondrial activity regulates the stemness, activation, proliferation, and metabolism of B-MSCs [31]. We examined the mitochondrial morphology and bioenergetics of B-MSCs from IPF patients and age-matched controls. Morphometric analysis of mitochondria in electronic microscope images showed a reduction in area and length in IPF B-MSCs consistent with mitochondrial fragmentation (Fig. 5a–c). Additionally, mitochondrial mass measured by MitoTracker staining was found to be increased in IPF B-MSCs (Fig. 5d). This suggests that, although smaller, mitochondria from IPF B-MSCs are more abundant than controls.

**Fig. 6:**
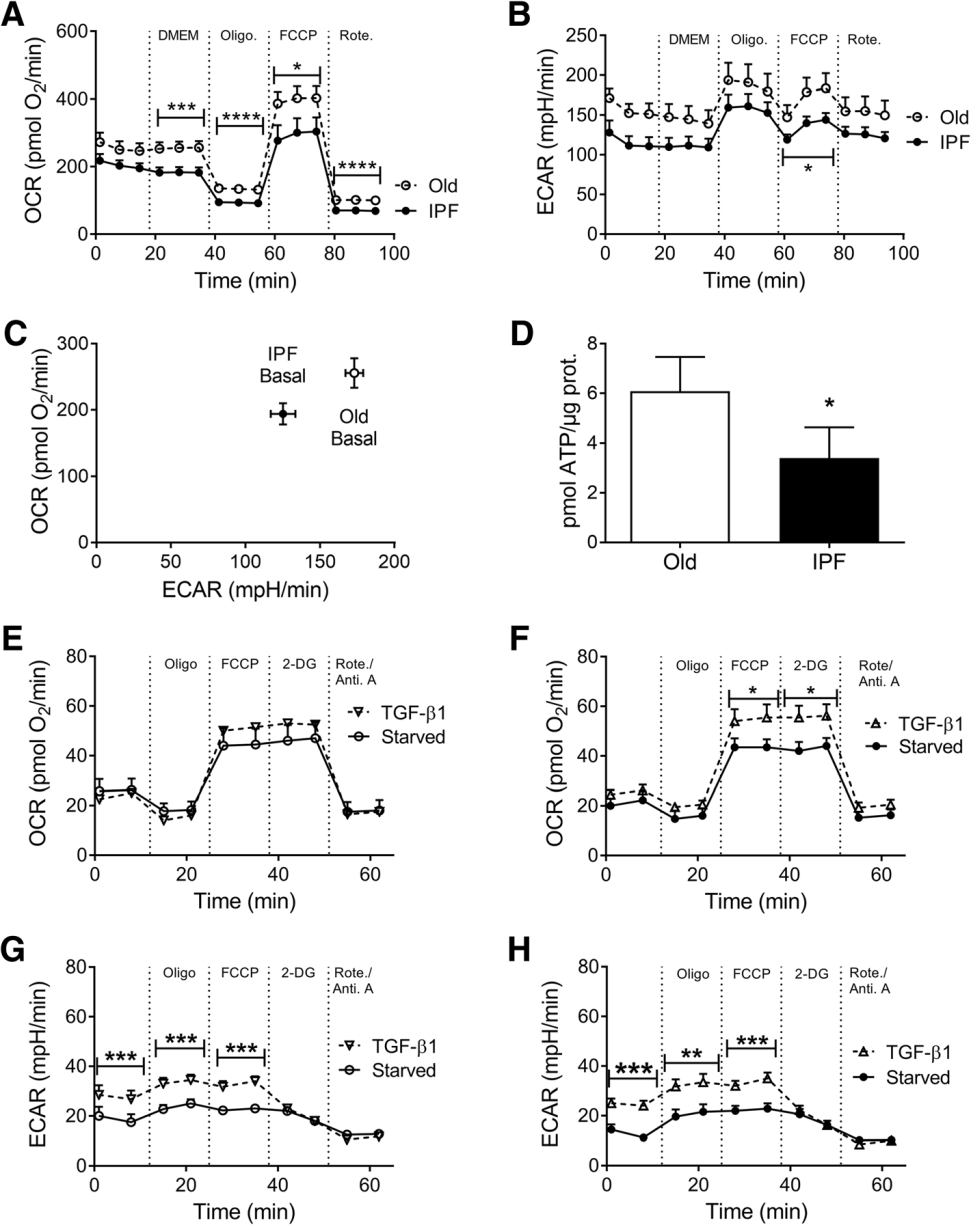
Mitochondria in B-MSCs from IPF patients have lower OCR and ECAR compared with aged-matched controls. Real-time measurements (mean ± SEM, *n* = 4 with technical triplicates) of the mitochondrial oxygen consumption rate (OCR; pmol O_2_/min) and extracellular acidification rate (ECAR; mpH/min) of B-MSC were measured under basal conditions and in response to the indicated mitochondrial inhibitors. OCR (**a**) and ECAR (**b**) in idiopathic pulmonary fibrosis (IPF) B-MSCs (continuous line) is significantly lower compared with B-MSC from age-matched controls (Old; dashed line). The basal relationship between OCR and ECAR (**c**) of IPF B-MSCs was lower compared with controls. Adenosine triphosphate (ATP) content in IPF B-MSCs (**d**) was measured by bioluminescence assay. Total ATP content was shown to be nonsignificantly lower in IPF B-MSCs compared with age-matched controls. To evaluate the effects of transforming growth factor (TGF)-β stimulation on B-MSCs from IPF and control patients, B-MSCs were stimulated with human recombinant activated TGF-β1 (5 ng/ml). Real-time measurements (mean ± SEM, *n* = 4 with technical triplicates) of the mitochondrial OCR and ECAR of B-MSCs were measured under basal conditions and in response to the indicated mitochondrial inhibitors. OCR in basal (continuous line) or TGF-β1-stimulated (dashed line) B-MSCs are shown for control (**e, g**) and IPF (**f, h**) patients; *p < 0.05, **p < 0.01, ***p < 0.001, ****p < 0.0001. 2-DG 2-deoxy-d-glucose, DMEM Dulbecco’s modified Eagle’s medium, FCCP carbonyl cyanide p-trifluoromethoxyphenylhydrazone, Oligo. oligomycin, Rote. rotenone

We evaluated the mitochondrial function by determination of bioenergetics profiles under basal conditions, as well as the respiratory rate after the injection of oligomycin, an inhibitor of the complex V of the electron transport chain (ETC), and maximal respiratory capacity on the injection of the mitochondrial inner membrane uncoupler carbonyl cyanide p-trifluoromethoxyphenylhydrazone (FCCP). Finally, the complex I inhibitor rotenone was administered to determine the nonmitochondrial oxygen consumption rate (OCR). IPF B-MSCs showed lower OCR at basal and maximal respiration conditions and after injection of mitochondrial complex inhibitors in comparison with control cells (Fig. 6a).

Metabolic reprogramming has been found in myofibroblasts from IPF patients. We analyzed glycolytic rates in IPF B-MSCs and controls. Extracellular acidification rate (ECAR) readings at baseline and after FCCP treatment were lower in IPF B-MSCs compared with controls, suggesting there was no glycolytic reprograming in these cells (Fig. 6b). In fact, when the relationship of basal OCR/ECAR was examined, IPF patients showed a less energetic phenotype compared with control individuals (Fig. 6c)**.** As a consequence of lower oxygen consumption and glycolytic rates, total adenosine triphosphate (ATP) content in IPF patients decreased compared with the control group (Fig. 6d).

TGF-β1 can stimulate OCR and ATP generation [32]. We studied the effects of TGF-β1 on oxidative phosphorylation after 4 h of TGF-β1 stimulation on IPF and control B-MSCs. Basal respiration was not affected by TGF-β1 stimulation in control and IPF cells. Compared with untreated (starved) cells, IPF B-MSCs stimulated with TGF-β1 showed significant increases in maximal respiration after FCCP treatment and in the presence of the glycolysis inhibitor 2-deoxy-d-glucose (2-DG) (Fig. 6f), whereas age-matched control B-MSCs did not show significant changes upon stimulation (Fig. 6e). These results suggest that IPF B-MSCs have a higher response to TGF-β1 stimulation upon uncoupling of the mitochondria and inhibition of glycolysis. ECAR bioprofiles showed a similar pattern in control (Fig. 6g) and IPF B-MSCs (Fig. 6h).

### Aged B-MSCs have a decreased capacity to prevent lung fibrosis progression

To assess changes in the capabilities of human B-MSCs to alter the severity of the lung injury, we evaluated the ability of age-matched control and IPF B-MSCs to prevent the development of bleomycin-induced lung fibrosis and then compared this with the response mediated by B-MSCs isolated from young donors. Two regimens of cell infusion were examined: a preventive regimen with infusion of cells 2 h after bleomycin injection, and a therapeutic regimen with cell infusion at day 7 post-bleomycin.

**Fig. 7:**
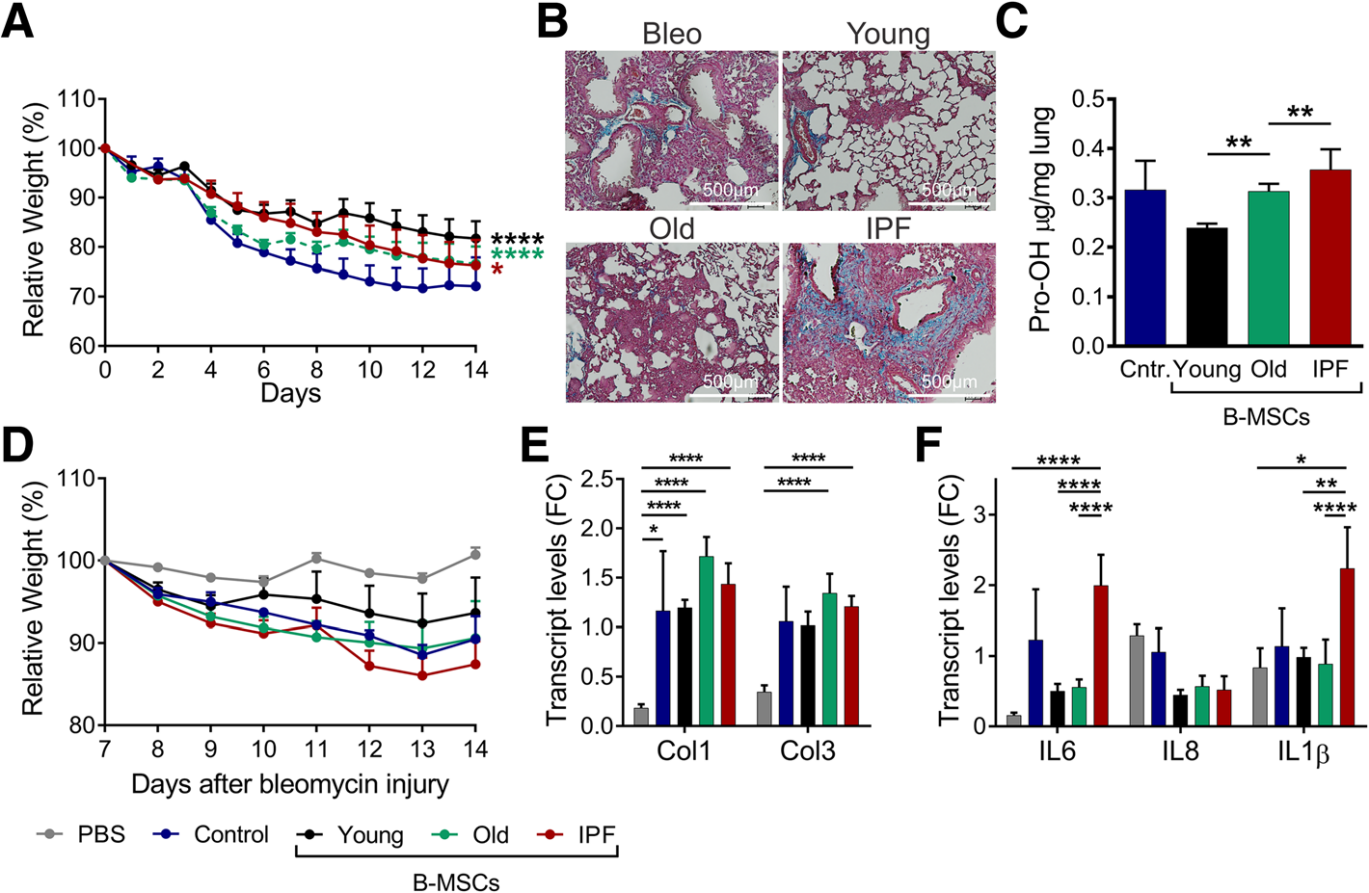
Aged B-MSCs have a decreased capacity to prevent lung fibrosis progression. C57BL/6 mice were subjected to bleomycin injury and subsequently treated with bone marrow-derived mesenchymal stem cells (B-MSCs) from young individuals (black), aged individuals (Old; green), idiopathic pulmonary fibrosis (IPF) patients (red) or cell medium (blue) intravenously at day 0 (**a–c**) or day 7 (**d–f**). **a, d** Percent of initial body weight curves are shown. The group that received B-MSCs from young donors presented lower weight loss compared with the bleomycin control group, and mice receiving B-MSCs from other groups (old and IPF patients) have a similar weight loss. Infusion of cells at day 7 show a higher weight loss when treated with IPF-MSCs (**d**); mean ± SEM; *n* = 5 in bleomycin control group and IPF B-MSC; *n* = 9 in young and old B-MSCs. **b** Masson’s trichrome staining of representative histologic sections (20×, scale bar = 100 μm). **c** Quantitation of hydroxyproline (Pro-OH) content in the lung. **e, f** In the group that received cells at day 7, mice treated with B-MSCs from IPF donors have higher levels of expression of profibrotic and proinflammatory genes compared with controls and young B-MSCs; *p < 0.05, **p < 0.01, ***p < 0.001, ****p < 0.0001. Cntr. control, Col collagen, FC fold-change, IL interleukin, PBS phosphate-buffered saline

Weight loss was used as a measurement of illness severity. As previously reported, mice in the preventive and therapeutic regimens that received young B-MSCs were protected against weight loss compared with bleomycin-treated mice without B-MSC infusion (Fig. 7a, d). Mice receiving old and IPF B-MSCs in the preventive regimen lost weight more severely than the mice infused with young B-MSCs, but less than mice in the bleomycin-alone control group (Fig. 7a). In the therapeutic regimen, old and IPF B-MSCs similarly failed to provide beneficial effects in bleomycin-injured mice, although IPF B-MSC mice had a substantial higher weight loss. Lung pathology was analyzed by Masson trichrome staining at day 14 post-bleomycin. Mice in the preventive regimen that received old and IPF B-MSCs developed extensive fibrosis similar to the bleomycin control group (Fig. 7b). In contrast, mice treated with young B-MSCs developed less lung fibrosis. Lung pathology findings correlated with collagen content measured by determination of hydroxyproline levels (Fig. 7c). In the therapeutic regimen, transcript levels of collagen 1 and 3 were similar in bleomycin-treated mice with or without B-MSC infusion (Fig. 7e). However, mice that received IPF B-MSCs showed significantly higher transcript levels of IL-6 and IL-1β (Fig. 7f).

**Fig. 8:**
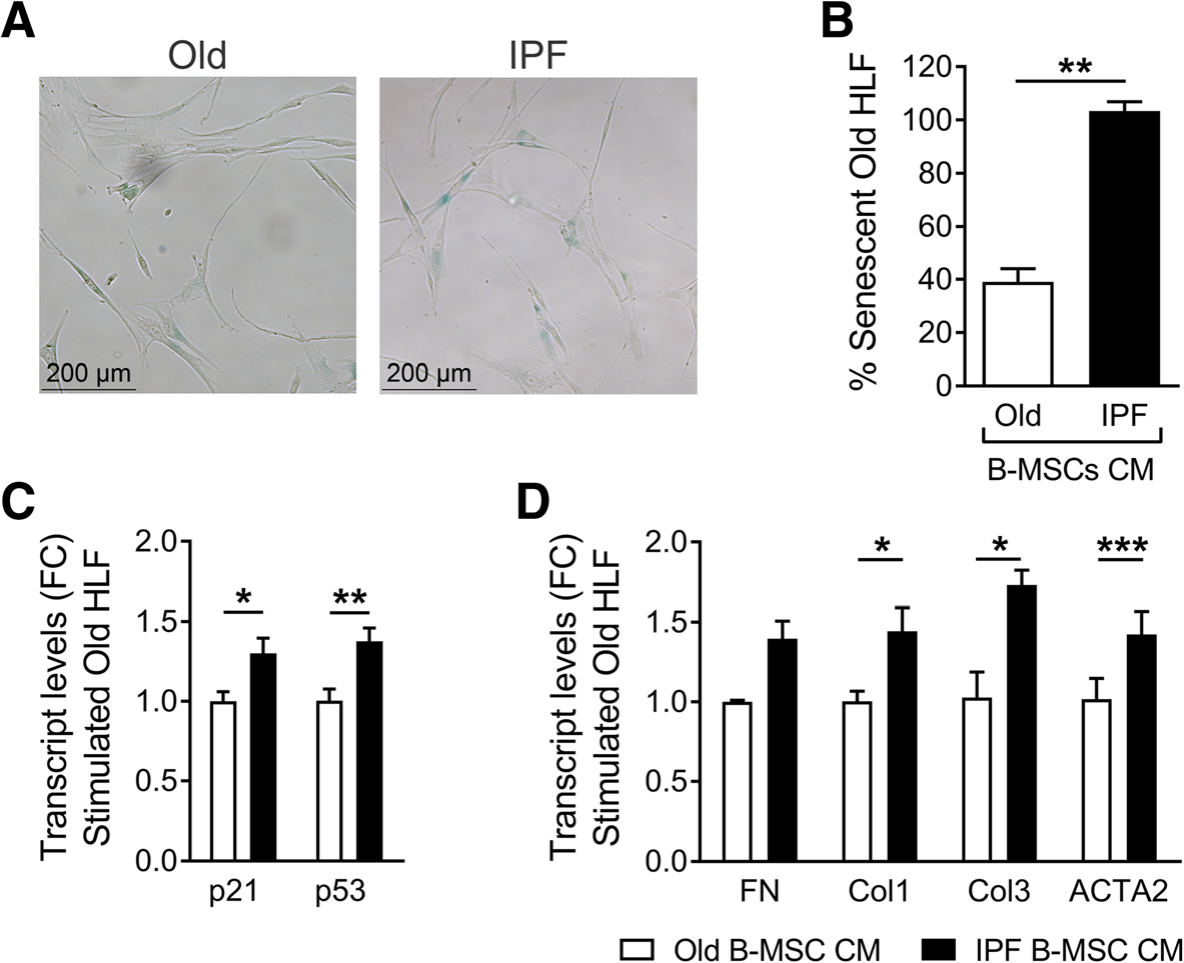
Stimulation of human lung fibroblasts (HLF) with bone marrow-derived mesenchymal stem cell (B-MSC) conditioned media (CM) from idiopathic pulmonary fibrosis (IPF) patients recapitulates senescence and fibrotic phenotypes in old HLF. HLF from a 65-year-old male were stimulated with CM from B-MSC of controls (Old; 73 ± 4 years of age) and IPF patients (72 ± 5 years of age) for 2 days. **a** HLF were subjected to SA-β-gal staining and percent of senescent cells were quantified (**b**). **c** mRNA expression levels in stimulated HLF were determined by qRT-PCR for senescent markers (p21 and p53) and **d** profibrotic SASPs (fibronectin (FN)1, collagen (Col)1, ACTA1, and Col3); mean ± SEM; *p ≤ 0.05, **p ≤ 0.01, ***p ≤ 0.001. Treatment with nonconditioned media does not increase transcript levels in HLF (data not shown). FC fold-change

To further study the potential profibrotic effect of IPF-MSCs, we analyzed whether conditioned media from IPF-MSCs change the phenotype of human lung fibroblasts. Aged human lung fibroblasts were cultured with conditioned media (CM) from IPF B-MSCs or age-matched controls. After 48 h of treatment, human lung fibroblasts showed increased expression of markers of senescence, including β-galactosidase activity (Fig. 8a, b), and upregulation of p16^INK4A^ and p53 (Fig. 8c). In parallel, higher expression of collagen 1, collagen 3, and fibronectin was found in fibroblasts cultured in the presence of CM from IPF B-MSCs (Fig. 8d).

## Discussion

IPF is an age-related systemic disease with a predominant lung phenotype. There is compelling evidence that, for unknown reasons, the lung can be the main target of systemic alterations such as telomere mutations, alterations in proteostasis, and mitochondrial dysfunction. It has been proposed that the reparative capacity of B-MSCs may be decreased with age [33]. In our current study, we have demonstrated that B-MSCs from IPF patients are defective when compared with age-matched controls. B-MSCs from IPF patients present mitochondrial dysfunction and impaired recovery capacity in response to in-vitro and in-vivo stimulation. In addition, B-MSCs from IPF patients showed evidence of DNA damage and a tendency to have telomere shortening. These findings clearly show that B-MSCs from IPF patients were more senescent than the age-matched controls.

Aging is a process that affects all cells, including mesenchymal stem cells. It has been suggested that, in aged mesenchymal stem cells and aged lungs, several pathways are altered that could increase the risk of IPF [33]. We have recently demonstrated that fibroblasts isolated from the lungs of IPF patients have an increase in markers of cell senescence [12]. However, there are limited data about the role of B-MSCs in IPF. In our original observation, using the murine model of bleomycin-induced lung fibrosis, we compared the effect of a single dose of intratracheal bleomycin in a model of accelerated aging on 6-month-old senescence-accelerated-prone (SAMP) mice and senescence-accelerated-resistant (SAMR) mice using 12-month-old mice [25]. Fourteen days after the insult, we observed a decrease in the ability to repair the lung in SAMP mice after bleomycin-induced lung injury, resulting in an increase in lung fibrosis when compared with SAMR mice. In SAMP mice, these changes were associated with higher levels of TGF-β1 in the lung and a decrease in the ability of B-MSCs to respond to the soluble signals of injury. In our current study using B-MSCs from control and IPF donors, we have observed that only animals treated with B-MSCs from young donors exhibit lower fibrosis after bleomycin injury, corroborating the fact that the capacity to respond to fibrosis is reduced in aged B-MSCs. This is in support of the findings observed in our cytokine studies. Although not significant, we observed a differential biological effect at 72 h of TGF-β1 stimulation in both groups. IPF B-MSCs cells had lower nonsignificant expression of TSG6 and KGF than old B-MSCs. This is concordant with our hypothesis, as both genes are associated with protective modulation of mesenchymal stem cells in lung fibrosis [34, 35].

A decrease in cell proliferation, mitochondrial dysfunction, telomere attrition, and cellular senescence are identified as hallmarks of aging [21]. Our results have globally demonstrated that B-MSCs from IPF patients show more advanced biological signs of aging compared with individuals of a similar age, which suggest that the hypothesis proposed by Selman and Pardo [7, 36] of IPF as an accelerated form of aging of the lung is also plausible in B-MSCs. In addition, these hallmarks relate to each other and could explain this accelerated process of aging in B-MSCs from IPF patients. We have found that, in IPF, B-MSCs have dysfunctional mitochondria with decreased OCR and ECAR compared with controls. On the other hand, dysfunctional mitochondria have been associated with a distinct senescent phenotype in human cells that results from an NADH-AMPK-p53-dependent pathway. Since the central role of mitochondria is to regulate cell function [8, 37], this could also be one of the factors contributing to an accentuated senescent phenotype in B-MSCs from IPF patients. However, other factors could also contribute to the induction of senescence. Dysfunctional telomeres and nontelomeric DNA damage may also transform the cell into a senescent phenotype [19]. In our study, despite the absence of a significant correlation in telomere shortening and IPF, we observed a tendency for a lower average telomere length consistent with that observed in other IPF studies with a higher sample size [38]. Thus, secondary to different factors, B-MSCs from IPF patients are more senescent, leading to a loss of the repair capacity, which as previously suggested could be one contributing cause to the development of IPF [33]. Additionally, correlating with the phenotype observed in IPF B-MSCs to the onset of the disease, a profibrotic phenotype was induced only in old lung fibroblasts.

In the present study, we have demonstrated that B-MSCs from IPF patients have important differences in mitochondrial function, increases in DNA damage that result in cell senescence, and defects in critical cell functions when compared with age-matched controls. IPF B-MSCs show signs of accelerated senescence that suggests a link between aging and the late onset of the disease. Given that MSCs exhibit decreased function with age and disease, this confirms the possible risk of the use of autologous stem cells in patients with IPF.

## Conclusions

MSCs, like other cells in IPF patients, have multiple defects that can result in an increase in the severity of the disease. We have identified extrapulmonary changes in the bone marrow-derived mesenchymal stem cells (B-MSCs). Although there is evidence in animal models, no human studies have assessed the function of IPF B-MSCs compared with age-matched old control donors. In our study, we demonstrate for the first time that B-MSCs from IPF patients are senescent with significant differences in mitochondrial function and accumulation of DNA damage resulting in defects in critical cell functions when compared with age-matched controls. Senescent IPF B-MSCs have the capability to stimulate paracrine senescence by inducing senescence in normal aged fibroblasts, suggesting a possible link between senescent B-MSCs and the late onset of the disease. Despite IPF being a disease with a respiratory phenotype and a major representation in the lung, our results show systemic consequences of the disease.

## Additional file

Additional file 1: Supplementary figures. (ZIP 623 kb)

## Abbreviations

2-DG: 2-Deoxy-d-glucose
ATP: Adenosine triphosphate
B-MSC: Bone marrow-derived mesenchymal stem cell
CM: Conditioned media
CORID: Committee for Oversight of Research and Clinical Training Involved Decedents
ECAR: Extracellular acidification rate
ETC: Electron transport chain
FCCP: Carbonyl cyanide p-trifluoromethoxyphenylhydrazone
IACUC: Institutional Animal Care and Use Committee
IL: Interleukin
IPF: Idiopathic pulmonary fibrosis
OCR: Oxygen consumption rate
SAMP: Senescence-accelerated-prone
SAMR: Senescence-accelerated-resistant
SASP: Senescence-associated secretory phenotype
SA-β-gal: Senescence-associated β-galactosidase
TGF: Transforming growth factor
