# Supplementary material for: Senescence of bone marrow-derived mesenchymal stem cells from patients with idiopathic pulmonary fibrosis
Source: Stem Cell Res Ther. 2018 Sep 26;9:257. doi: 10.1186/s13287-018-0970-6 (PMC6158816; doi:10.1186/s13287-018-0970-6)
Supplement: Supplementary file 1 — Supplementary figures. (ZIP 623 kb) [file 13287_2018_970_MOESM1_ESM.zip › FINAL IPF MSC_SCRT-caption.docx]

**Senescence of bone marrow-derived mesenchymal stem cells from patients with idiopathic pulmonary fibrosis**

Nayra Cárdenes^1,2^, Diana Álvarez ^1,2^, Jacobo Sellarés^1,2,3^, Yating Peng^1,2,4^, Catherine Corey^2,5^, Sophie Wecht^1,2^, Seyed Mehdi Nouraie^1,2^, Swaroop Shanker^1,2^, John Sembrat^1,2^, Marta Bueno^2,5^, Sruti Shiva^5,6^, Ana L. Mora^2,5^ and Mauricio Rojas^1,2,5^

^1^Dorothy P. & Richard P. Simmons Center for Interstitial Lung Disease, University of Pittsburgh School of Medicine, Pittsburgh, PA, USA.

^2^Division of Pulmonary, Allergy and Critical Care Medicine, University of Pittsburgh School of Medicine, Pittsburgh, PA, USA.

^3^Interstitial Lung Disease Program, Hospital Clínic, Barcelona, Spain

^4^Research Unit of Respiratory Diseases, Central South University, Changsha Hunan, 410011, China.

^5^Vascular Medicine Institute of the University of Pittsburgh, University of Pittsburgh School of Medicine, Pittsburgh, PA, USA.

^6^Department of Pharmacology & Chemical Biology, University of Pittsburgh

**Emails:** NC: [cardenesn@upmc.edu](mailto:cardenesn@upmc.edu), DA: [dma56@pitt.edu](mailto:dma56@pitt.edu), JS: [sellares@clinic.cat](mailto:sellares@clinic.cat), YP: [pengyating2011@126.com](mailto:pengyating2011@126.com), CC: [cgc9@pitt.edu](mailto:cgc9@pitt.edu), SW: [sophiewecht@gmail.com](mailto:sophiewecht@gmail.com), SMN: [nouraies@upmc.edu](mailto:nouraies@upmc.edu), SS: [swaroop.shankar@gmail.com](mailto:swaroop.shankar@gmail.com), JS: [sembratjc@upmc.edu](mailto:sembratjc@upmc.edu), MB: [mbueno@pitt.edu](mailto:mbueno@pitt.edu), SS: [sss43@pitt.edu](mailto:sss43@pitt.edu), ALM: [anamora@pitt.edu](mailto:anamora@pitt.edu), MR:rojasm@upmc.edu

**Running Title:** IPF mesenchymal stem cells.

Author for correspondence: Mauricio Rojas W1244 BST Tower 200 Lothrop Street Pittsburgh PA 15261. [rojasm@upmc.edu](mailto:rojasm@upmc.edu).

**ABSTRACT**

*Background:* Idiopathic pulmonary fibrosis (IPF) is a chronic lung disease in which age is the most important risk factor. Different mechanisms associated with aging including stem cell dysfunction have been described to participate in the pathophysiology of IPF. We observed an extra-pulmonary effect associated with IPF, increase in cell senescence of bone marrow-derived mesenchymal stem cells (B-MSCs).

*Methods:* B-MSCs were obtained from vertebral bodies procured from IPF patients and age-matched normal controls. Cell senescence was determined by cell proliferation, expression of markers of cell senescence p16^INK4A^, p21 and β-gal activity. Mitochondrial function and DNA damage were measured. Paracrine induction of senescence and pro-fibrotic responses were analyzed *in vitro* using human lung fibroblasts. The reparative capacity of B-MSCs was examined *in vivo* using the bleomycin induced lung fibrosis model.

*Results:* In our study, we demonstrate for the first time that B-MSCs from IPF patients are senescent with significant differences in mitochondrial function, with accumulation of DNA damage resulting in defects in critical cell functions when compared to age-matched controls. Senescent IPF B-MSCs have the capability of paracrine senescence by inducing senescence in normal aged fibroblasts, suggesting a possible link between senescent B-MSCs and the late onset of the disease. IPF B-MSCs also showed a diminished capacity to migrate and were less effective in preventing fibrotic changes observed in mice after bleomycin-induced injury, increasing illness severity and pro-inflammatory responses.

*Conclusions:* We are describing extra-pulmonary alterations of B-MSCs from IPF patients. The consequences of having senescent B-MSCs are not completely understood, but the decrease on their ability to respond to normal activation and the risk of having a negative impact on the local niche by inducing inflammation and senescence in the neighboring cells can suggest a new link between B-MSC and the onset of the disease.

Keywords: Idiopathic pulmonary fibrosis, mesenchymal stem cells, aging, cellular senescence, and mitochondria.

**BACKGROUND**

Idiopathic pulmonary fibrosis (IPF) is a chronic interstitial lung disease characterized by a progressive and irreversible loss of lung function by accumulation of scar tissue (1-3). Its annual incidence in the USA has been estimated to be 6.8-16.3 cases per 100,000 inhabitants (1, 4). IPF has a heterogeneous evolution, and even though periods of clinical stability may be observed, progressive deterioration is unavoidable with a median survival of 3-5 years from the time of diagnosis (5). Although two new approved therapies are currently available (pirfenidone and nintedanib), their efficacy is limited and several adverse effects have been described (6).

Aging is considered the main risk factor for IPF (7-11). Along with others, we have demonstrated that there is an increase of markers of cell senescence in lung fibroblasts from IPF patients (12-15). Additionally, we have shown that in animal models of lung injury, that aged bone marrow-derived mesenchymal stem cells (B-MSCs) have decreased protecting activity (16). This is in contrast to what we had previously described in young animal models of pulmonary fibrosis, where infusion of B-MSCs isolated from normal young donors in the initial stages of the injury results in a decrease of collagen deposition in the lung after bleomycin instillation (17, 18). Therefore, we aimed to determine the differences in the biological and functional characteristics of B-MSCs from healthy individuals and IPF patients within the same age range. Characterization of IPF B-MSCs shows an increase of cell senescence linked to an upsurge of senescence-associated secretory phenotype, or SASPs, promoting a pro-inflammatory milieu and increasing deposition of components from the extracellular matrix. Our data suggests that extrapulmonary alterations in B-MSCs from IPF patients might contribute to the pathogenesis of the disease. To our knowledge, this is the first report describing amelioration in functional and reparative capacities of the endogenous non-pulmonary MSCs from patients who have developed IPF.

**METHODS**

(See online data supplement for detailed methods)

Human B-MSCs isolation and manipulation

Human B-MSCs isolation was approved by the Committee for Oversight of Research and Clinical Training Involved Decedents (CORID) of the University of Pittsburgh. As previously described, B-MSCs were isolated from bone marrow fragments from cadaveric vertebral bones (16). B-MSCs were divided into 3 groups: young donors (18-30 yr; n=7), old donors (57-82 yr; n=11) and IPF patients (60-82 yr; n=8) (**Supp. Table 1**). B-MSCs were isolated, cultured and expanded according to previously published protocols (**see online data supplement**).

Animals and animal treatment

Female 11-week C57BL/6 mice (The Jackson Laboratory, Bar Harbor, ME) were treated with 2 U/Kg of bleomycin hydrochloride solution (63323-136-10, APP pharmaceuticals, Schaumburg, IL dissolved to 1 U/ml in sterile saline), delivered by direct injection into the trachea using a 0.9 mm needle. While under anesthesia, a cell suspension of 500,000 human B-MSC in 100 µL of culture medium was injected intravenously. Control group mice received the same volume of sterile media solution (**see online data supplement for details**). All animal protocols were reviewed and approved by the Institutional Animal Care and Use Committee (IACUC).

Statistical Analysis

Statistical analyses were done using Graph Pad Prism version 7 (GraphPad Software) and STATA version 13 (Stata Corporation). Comparisons between control and IPF B-MSCs were made using Mann-Whitney test. Kruskal-Wallis and Dunn tests were used for between group comparisons. For time-dependent observations, we used mixed effect models with a robust variance estimator to calculate the difference between groups in experiment outcomes.

**RESULTS**

IPF B-MSCs are more senescent than age-match control B-MSCs

B-MSCs isolated from IPF patients showed morphological changes characterized by increased cell size accompanied by replicative senescence in comparison with B-MSCs from age-match controls. Quantification of B-MSCs proliferation by measurement of DNA staining showed decreased cell proliferation (**Fig. 1A**), which was confirmed by determination of flow cytometric quantification of cell cycle phases. Lower number of IPF B-MSCs was observed in G2/M phase compared to old control B-MSCs and higher number of IPF-B-MSCs were found in G0 phase after TGF-β1 stimulation (**Supplementary Fig. 1**).

To confirm the senescence phenotype of B-MSCs from IPF patients, we investigated the presence of other markers of senescence. Measurements of β­galactosidase (SA-β-gal) activity showed significantly higher positivity in B-MSCs derived from IPF patients compared to the control group (**Fig. 1B-C**). Furthermore, transcript levels of the inhibitor of cell cycle p21 was significantly increased in IPF B-MSCs and moderately increased in p16^INK4A^ and p53 when compared with age-match controls (19, 20) (**Fig. 1D**).

DNA damage in B-MSCs from IPF patients

The ability to repair DNA declines with age, and the consequent accumulation of DNA damage leads the cells to senescence or apoptosis (21). We evaluated DNA damage in B-MSCs from IPF patients and controls by determination of γ-H2AX phosphorylation (**Fig. 2A**). Quantification of positive cells showed a significantly higher percentage of IPF B-MSCs with DNA damage compared to age-matched controls (**Fig. 2B**). Additionally, telomere shortening has been identified as one of the hallmarks of aging (21). We observed an important tendency to a shorter telomere length in B-MSCs from IPF patients when measured by flowFISH (22) (**Supplementary Fig. 2**) that correlates with a senescent phenotype.

Decrease stemness and function of IPF B-MSCs

B-MSCs are characterized by their ability to differentiate into a chondriocytes, osteocytes and adipocytes (18, 23, 24). With age, the differentiation potential is attenuated or inhibited for chondriogenesis and osteogenesis. Conversely, the differentiation potential into the adipogenic lineage is increased with senescence (25). We evaluated the ability of B-MSCs to differentiate by treating them with adipogenic media. After 21 days of treatment, quantification of Oil-Red staining demonstrated a significant decrease of positive cells in cell cultures of IPF B-MSCs compared with the age-match control, suggesting that the differentiation capacity was diminished in IPF-B-MSCs (**Fig. 3**)**.**

TGF-β1 also plays an important role in directing fate decision in B-MSCs and modulating regenerative function of B-MSCs (26-28). We analyzed the response to TGF-β1 stimulation on control and IPF B-MSCs at 24h and 72h. At 24h, IPF-MSCs expressed higher levels of IL-6 (a well-known factor of senescence associated secretory phenotype) than compared to control cells (data not shown). In sharp contrast, at 72h of TGF-β1 stimulation, transcript levels of growth factors associated with wound healing and reduction of tissue fibrosis such as TGS-6, KGF and IL-1RN were diminished in IPF-B-MSCs (data not shown).

The wound healing process is also affected by the capacity of B-MSCs to migrate to the injured organ (16). Using a combination of parabiosis and bleomycin induced lung fibrosis models, we first analyzed the effect of aging for *in vivo* migration of B-MSCs (see **online data supplement**). We found that bleomycin-injured lungs, independent of the age, can generate the appropriate signals which promote the recruitment of cells that express a pattern of surface markers that resemble B-MSCs into the lung. However, only B-MSCs from young mice were able to migrate and home into the injured lung, suggesting an age-related defect of B-MSCs to respond to chemotactic stimulus (**Supplementary Fig. 3**). Secondly, *in vitro* studies were used to analyze the migration capacity of IPF-B-MSCs. Migration and proliferation of IPF B-MSCs and controls were determined by *in vitro* wound closure assays. Control B-MSCs were able to close the wound after 48 hours of stimulation with 1% of serum from IPF patients. Contrarily, IPF B-MSCs failed to close the wound with same stimuli (**Fig. 4**). Both control and IPF-B-MSCs have minimal migration after 24h of TGF-β1 stimulation or media without serum (data not shown).

IPF B-MSCs have fragmented and dysfunctional mitochondria

Mitochondrial dysfunction has been implicated in the induction of cellular senescence and fibrosis (8, 29, 30). It is also recognized that mitochondrial activity regulates the stemness, activation, proliferation, and metabolism of B-MSCs (31). We examined the mitochondrial morphology and bioenergetics of B-MSCs from IPF patients and age-matched controls. Morphometric analysis of mitochondria in electronic microscope images showed in IPF B-MSCs a reduction in area and length consistent with mitochondrial fragmentation (**Fig. 5A-C**). Additionally, mitochondrial mass measured by MitoTracker staining was found to be increased in IPF B-MSCs **(Fig. 5D)**. This suggests that, although smaller, mitochondria from IPF B-MSCs are more abundant than controls.

We evaluated the mitochondrial function by determination of bioenergetics profiles at basal conditions, as well as the respiratory rate on the injection of oligomycin, an inhibitor of the complex V of the electron transport chain (ETC), and maximal respiratory capacity on the injection of the mitochondrial inner membrane uncoupler carbonyl cyanine p-trifuormethoxyphenylhydrazone (FCCP). Finally, the complex I inhibitor rotenone was administered to determine the non-mitochondrial oxygen consumption rate (OCR). IPF B-MSCs showed lower OCR at basal and maximal respiration conditions and after injection of mitochondrial complex inhibitors in comparison to control cells (**Fig. 6A**).

Metabolic reprogramming has been found in myofibroblasts from IPF patients. We analyzed glycolytic rates in IPF B-MSCs and controls. Baseline and after FCCP treatment extracellular acidification rate (ECAR) readings were lower in IPF B-MSCs compared to controls suggesting there was not glycolytic reprograming in these cells (**Fig. 6B**). In fact, when the relationship of basal OCR/ECAR was examined, IPF patients showed a less energetic phenotype compared to control individuals (**Fig. 6C**)**.** As a consequence of lower oxygen consumption and glycolytic rates, total ATP content in IPF patients decreased compared to the control group (**Fig. 6D**).

TGF-β1 can stimulate OCR and ATP generation (32). We studied the effects of TGF-β1 on oxidative phosphorylation after 4 hours of TGF-β1 stimulation on IPF and control B-MSCs. Basal respiration was not affected by TGF-β1 stimulation in control and IPF cells. Compared to untreated cells (starved), IPF B-MSCs stimulated with TGF-β1 showed significant increase on maximal respiration after FCCP treatment and in the presence of the glycolysis inhibitor (2-DG) (**Fig. 6F**), primero F que E?? whereas age-matched control B-MSCs didn’t show significant changes upon stimulation (**Fig. 6E**). These results suggest that IPF B-MSCs have a higher response to TGF-β1 stimulation upon uncoupling of the mitochondria and inhibition of glycolysis. ECAR bioprofiles showed a similar pattern in control (**Fig. 6G**) and IPF B-MSCs (**Fig. 6H**).

Aged B-MSCs have a decreased capacity to prevent lung fibrosis progression

To assess changes in the capabilities of human B-MSCs to alter the severity of the lung injury, we evaluated the ability of age-match control and IPF B-MSCs to prevent the development of bleomycin-induced lung fibrosis and then compared to the response mediated by B-MSCs isolated from young donors. Two regimens of cell infusion were examined: a preventive regimen with infusion of cells two hours after bleomycin injection, and a therapeutic regime with cell infusion at day 7 post-bleomycin.

Weight loss was used as a measurement of illness severity. As previously reported, mice in the preventive and therapeutic regiment that received young B-MSCs were protected against weight loss compared to bleomycin treated mice without B-MSC infusion (**Fig. 7A, 7D**). Mice receiving old and IPF B-MSCs in the preventive regime loss weight more severely than the mice infused with young B-MSCs but less than mice in the bleomycin alone control group (**Fig. 7A**). In the therapeutic regiment, old and IPF B-MSCs similarly failed to provided beneficial effect in bleomycin injured mice, although IPF B-MSCs had a substantial higher weight loss. Lung pathology was analyzed by Masson Trichrome staining at day 14 post-bleomycin. Mice in the preventive regime that received old and IPF B-MSCs developed extensive fibrosis similar to the bleomycin control group (**Fig. 7B**). In contrast, mice treated with young B-MSCs developed less lung fibrosis. Lung pathology findings correlated with collagen content measured by determination of hydroxiproline levels (**Fig. 7C**). In the therapeutic regime, transcript levels of collagen 1 and 3 were similar in bleomycin treated mice with or without B-MSC infusion (**Fig. 7E**). However, mice that received IPF B-MSCs showed significantly higher transcript levels of IL-6 and IL-1β (**Fig. 7F**).

To further study the potential profibrotic effect of IPF-MSCs, we analyzed whether conditioned media from IPF-MSCs change the phenotype of human lung fibroblasts. Aged human lung fibroblasts were cultured with conditioned media (CM) from IPF B-MSCs or age-match controls. After 48h of treatment, human lung fibroblasts showed increased expression of markers of senescence including β-galactosidase activity (**Fig. 8A-B**), and upregulation of p16^INK4A^ and p53 (**Fig. 8C**). In parallel, higher expression of collagen 1, collagen 3, and fibronectin was found in fibroblasts culture in the presence of CM from IPF-B-MSCs (**Fig. 8D**).

**DISCUSSION**

IPF is an age-related systemic disease with a predominant lung phenotype. There is compelling evidence that for unknown reasons, the lung can be the main target of systemic alterations such as telomere mutations, alterations in proteostasis, and mitochondrial dysfunction. It has been proposed that the reparative capacity of B-MSCs may be decreased with age (33). In our current study, we have demonstrated that B-MSCs from IPF patients are defective when compared with age-matched controls. B-MSCs from IPF patients present mitochondrial dysfunction and impaired recovery capacity in response to *in vitro* and *in vivo* stimulation. In addition, B-MSCs from IPF patients showed evidence of DNA damage and a tendency to have telomere shortening. These findings clearly show that B-MSCs from IPF patients were more senescent than the age-matched controls.

Aging is a process that affects all cells, including mesenchymal stem cells. It has been suggested that, in aged mesenchymal stem cells and aged lungs, several pathways are altered that could increase the risk of IPF (33). We have recently demonstrated that fibroblasts isolated from the lungs of IPF patients have an increase in markers of cell senescence (12). However, there is limited data about the role of B-MSCs in IPF. In our original observation, using the murine model of bleomycin-induced lung fibrosis, we compared the effect of a single dose of intratracheal bleomycin in a model of accelerated aging on 6-month-old senescence-accelerated-prone mice (SAMP) and SA-resistant mice (SAMR) with 12 month-old mice (25). Fourteen days after the insult, we observed a decrease in the ability to repair the lung in SAMP after bleomycin-induced lung injury, resulting in an increase in lung fibrosis when compared to SAMR. In SAMP, these changes were associated with higher levels of TGF-β1 in the lung and a decrease in the ability of B-MSCs to respond to the soluble signals of injury. In our current study using B-MSCs from control and IPF donors, we have observed that only animals treated with B-MSCs from young donors exhibit lower fibrosis after bleomycin injury, corroborating the fact that the capacity to respond to fibrosis is reduced in aged B-MSCs. This is in support with the findings observed in our cytokine studies. Although not significant, we observed a differential biological effect at 72 hours of TGF-β1 stimulation in both groups. IPF B-MSCs cells had lower non-significant expression of TSG6 and KGF than old B-MSCs. This is concordant with our hypothesis, as both genes are associated with protective modulation of mesenchymal stem cells in lung fibrosis (34, 35).

Decrease in cell proliferation, mitochondrial dysfunction, telomere attrition, and cellular senescence are identified as hallmarks of aging (21). Our results have globally demonstrated that B-MSCs from IPF patients show more advanced biological signs of aging compared to individuals of similar age, which suggest that the hypothesis proposed by Selman *et al*. (7, 36) of IPF as an *accelerated* form of aging of the lung is also plausible in B-MSCs. In addition, these hallmarks relate to each other and could explain this *accelerated* process of aging in B-MSCs from IPF patients. We have found that, in IPF, B-MSCs have dysfunctional mitochondria with decreased OCR and ECAR compared to controls. On the other hand, dysfunctional mitochondria have been associated with a distinct senescent phenotype in human cells that results from an NADH-AMPK-p53 dependent pathway. Since the central role of mitochondria is to regulate cell function (8, 37), this could also be one of the factors contributing to an accentuated senescent phenotype in B-MSCs from IPF patients. However, other factors could also contribute to the induction of senescence. Dysfunctional telomeres and non-telomeric DNA damage may also transform the cell into a senescent phenotype (19). In our study, despite the absence of a significant correlation in telomere shortening and IPF, we observed a tendency for a lower average telomere length consistent to what is observed in other IPF studies with higher sample size (38). Thus, secondary to different factors, B-MSCs from IPF patients are more senescent, leading to a loss in the repair capacity, which as previously suggested could be one contributing cause to the development of IPF (33). Additionally, correlating with the phenotype observed in IPF-B-MSC to the onset of the disease, a profibrotic phenotype was induced only in old lung fibroblasts.

In the present study, we have demonstrated that B-MSCs from IPF patients have important differences in mitochondrial function, increase in DNA damage that result in cell senescence, and defects in critical cell functions when compared to age-matched controls. IPF B-MSCs show signs of accelerated senescence that suggest a link between aging and the late onset of the disease. Given that MSCs exhibit decreased function with age and disease confirms the possible risk of the use of autologous stem cells in patients with IPF.

**CONCLUSIONS**

MSCs, like other cells in IPF patients, have multiple defects that can result on the increase in the severity of the disease. We have identified extra-pulmonary changes in the bone marrow-derived mesenchymal stem cells (B-MSCs). Although there is evidence in animal models, no human studies have assessed the function of IPF B-MSCs compared to age-matched old control donors. In our study, we demonstrate for the first time that B-MSCs from IPF patients are senescent with significant differences in mitochondrial function, accumulation of DNA damage resulting on defects in critical cell functions when compared to age-matched controls. Senescent IPF B-MSCs have the capability to stimulate paracrine senescence by inducing senescence in normal aged fibroblasts, suggesting a possible link between senescent B-MSCs and the late onset of the disease. Despite IPF being a disease with a respiratory phenotype and a major representation in the lung, our results show systemic consequences of the disease.

**List of abreviations**

IPF: Idiopatic pulmonary fibrosis

B-MSCs: Bone marrow-derived mesenchymal stem cells.

SA-β-gal: Senescence associated β-galactosidase

SASP: Senescence-associated secretory phenotype

CORID: Committee for oversight of research and clinical training involved decedents

IACUC: Institutional Animal Care and Use Committee

ETC: Electron transport chain

FCCP: Carbonyl cyanine p-trifuormethoxyphenylhydrazone

OCR: Oxygen consumption rate

ECAR: Extracellular acidification rate

ATP: Adenosine triphosphate

2-DG: 2-Deoxy-D-glucose

CM: Conditioned media

SAMP: Senescence-accelerated-prone

SAMR: SA-resistant mice

HLF: Human lung fibroblasts

**DECLARATIONS**

Ethics approval and consent to participate

Human B-MSCs isolation was approved by the Committee for Oversight of Research and Clinical Training Involved Decedents (CORID) of the University of Pittsburgh (#101 and #451).

All animal protocols were reviewed and approved by the Institutional Animal Care and Use Committee (IACUC), protocol number 16088577.

Consent for publication

Not applicable.

Availability of data and material

The datasets used and/or analyzed during the current study are available from the corresponding author on reasonable request.

Competing interests

The authors declare that they have no competing interests.

Funding

NIH R01HLI123766 (MR), R01HL119476 (AM)

Authors' contributions

N.C.: Conception and Design, provision of study material or patients, collection and/or assembly of data, Data analysis and interpretation, Manuscript writing, final approval of manuscript.

D.Á.: collection and/or assembly of data, Data analysis and interpretation, final approval of manuscript.

J.S.: collection and/or assembly of data, Data analysis and interpretation, Manuscript writing, final approval of manuscript.

Y.P.: collection and/or assembly of data, final approval of manuscript

C.C.: collection and/or assembly of data, final approval of manuscript.

S.W.: collection and/or assembly of data, final approval of manuscript.

SM.N.: Sample size calculation and statistical analysis of all the experiments.

S.S.: collection and/or assembly of data, final approval of manuscript.

J.S.: provision of patient samples, final approval of manuscript.

M.B.: Data analysis and interpretation, final approval of manuscript.

S.S.: Data analysis and interpretation, final approval of manuscript.

A.M.: Data analysis and interpretation, final approval of manuscript.

M.R.: Conception and Design, provision of study material or patients, collection and/or assembly of data, Data analysis and interpretation, Manuscript writing, final approval of manuscript.

Acknowledgements

The authors wish to thank Chandler Caufield and Jordan Bullock (Department of Medicine, Division of Pulmonary, Allergy, and Critical Care Medicine; University of Pittsburgh Medical Center; Pittsburgh, PA; USA) for their assistance in editing.

Authors' information (optional)

Not applicable.

**REFERENCES**

1. Raghu G, Weycker D, Edelsberg J, Bradford WZ, Oster G. Incidence and prevalence of idiopathic pulmonary fibrosis. *American journal of respiratory and critical care medicine* 2006; 174: 810-816.

2. Raghu G, Rochwerg B, Zhang Y, Garcia CA, Azuma A, Behr J, Brozek JL, Collard HR, Cunningham W, Homma S, Johkoh T, Martinez FJ, Myers J, Protzko SL, Richeldi L, Rind D, Selman M, Theodore A, Wells AU, Hoogsteden H, Schunemann HJ, American Thoracic S, European Respiratory s, Japanese Respiratory S, Latin American Thoracic A. An Official ATS/ERS/JRS/ALAT Clinical Practice Guideline: Treatment of Idiopathic Pulmonary Fibrosis. An Update of the 2011 Clinical Practice Guideline. *American journal of respiratory and critical care medicine* 2015; 192: e3-19.

3. Martinez FJ, Collard HR, Pardo A, Raghu G, Richeldi L, Selman M, Swigris JJ, Taniguchi H, Wells AU. Idiopathic pulmonary fibrosis. *Nat Rev Dis Primers* 2017; 3: 17074.

4. Duchemann B, Annesi-Maesano I, Jacobe de Naurois C, Sanyal S, Brillet PY, Brauner M, Kambouchner M, Huynh S, Naccache JM, Borie R, Piquet J, Mekinian A, Virally J, Uzunhan Y, Cadranel J, Crestani B, Fain O, Lhote F, Dhote R, Saidenberg-Kermanac'h N, Rosental PA, Valeyre D, Nunes H. Prevalence and incidence of interstitial lung diseases in a multi-ethnic county of Greater Paris. *Eur Respir J* 2017; 50.

5. Ahluwalia N, Shea BS, Tager AM. New therapeutic targets in idiopathic pulmonary fibrosis. Aiming to rein in runaway wound-healing responses. *American journal of respiratory and critical care medicine* 2014; 190: 867-878.

6. Raghu G, Selman M. Nintedanib and pirfenidone. New antifibrotic treatments indicated for idiopathic pulmonary fibrosis offer hopes and raises questions. *American journal of respiratory and critical care medicine* 2015; 191: 252-254.

7. Selman M, Pardo A. Revealing the pathogenic and aging-related mechanisms of the enigmatic idiopathic pulmonary fibrosis. an integral model. *American journal of respiratory and critical care medicine* 2014; 189: 1161-1172.

8. Mora AL, Bueno M, Rojas M. Mitochondria in the spotlight of aging and idiopathic pulmonary fibrosis. *The Journal of clinical investigation* 2017; 127: 405-414.

9. Mora AL, Rojas M. Aging and lung injury repair: a role for bone marrow derived mesenchymal stem cells. *J Cell Biochem* 2008; 105: 641-647.

10. Mora AL, Rojas M. Chair's Summary: Lung Aging and Regeneration. 2016 Transatlantic Airway Conference. *Ann Am Thorac Soc* 2016; 13: S396-S397.

11. Mora AL, Rojas M, Pardo A, Selman M. Emerging therapies for idiopathic pulmonary fibrosis, a progressive age-related disease. *Nat Rev Drug Discov* 2017; 16: 755-772.

12. Alvarez D, Cardenes N, Sellares J, Bueno M, Corey C, Hanumanthu VS, Peng Y, D'Cuhna H, Sembrat J, Nouraie M, Shanker S, Caufield C, Shiva S, Armanios M, Mora AL, Rojas M. IPF lung fibroblasts have a senescent phenotype. *Am J Physiol Lung Cell Mol Physiol* 2017: ajplung 00220 02017.

13. Yanai H, Shteinberg A, Porat Z, Budovsky A, Braiman A, Ziesche R, Fraifeld VE. Cellular senescence-like features of lung fibroblasts derived from idiopathic pulmonary fibrosis patients. *Aging* 2015; 7: 664-672.

14. Yanai H, Fraifeld VE. The role of cellular senescence in aging through the prism of Koch-like criteria. *Ageing Res Rev* 2017; 41: 18-33.

15. Schafer MJ, White TA, Iijima K, Haak AJ, Ligresti G, Atkinson EJ, Oberg AL, Birch J, Salmonowicz H, Zhu Y, Mazula DL, Brooks RW, Fuhrmann-Stroissnigg H, Pirtskhalava T, Prakash YS, Tchkonia T, Robbins PD, Aubry MC, Passos JF, Kirkland JL, Tschumperlin DJ, Kita H, LeBrasseur NK. Cellular senescence mediates fibrotic pulmonary disease. *Nat Commun* 2017; 8: 14532.

16. Bustos ML, Huleihel L, Kapetanaki MG, Lino-Cardenas CL, Mroz L, Ellis BM, McVerry BJ, Richards TJ, Kaminski N, Cerdenes N, Mora AL, Rojas M. Aging mesenchymal stem cells fail to protect because of impaired migration and antiinflammatory response. *American journal of respiratory and critical care medicine* 2014; 189: 787-798.

17. Ortiz LA, Gambelli F, McBride C, Gaupp D, Baddoo M, Kaminski N, Phinney DG. Mesenchymal stem cell engraftment in lung is enhanced in response to bleomycin exposure and ameliorates its fibrotic effects. *Proc Natl Acad Sci U S A* 2003; 100: 8407-8411.

18. Rojas M, Xu J, Woods CR, Mora AL, Spears W, Roman J, Brigham KL. Bone marrow-derived mesenchymal stem cells in repair of the injured lung. *Am J Respir Cell Mol Biol* 2005; 33: 145-152.

19. Campisi J, d'Adda di Fagagna F. Cellular senescence: when bad things happen to good cells. *Nat Rev Mol Cell Biol* 2007; 8: 729-740.

20. Calio A, Zamo A, Ponzoni M, Zanolin ME, Ferreri AJ, Pedron S, Montagna L, Parolini C, Fraifeld VE, Wolfson M, Yanai H, Pizzolo G, Doglioni C, Vinante F, Chilosi M. Cellular Senescence Markers p16INK4a and p21CIP1/WAF Are Predictors of Hodgkin Lymphoma Outcome. *Clin Cancer Res* 2015; 21: 5164-5172.

21. Lopez-Otin C, Blasco MA, Partridge L, Serrano M, Kroemer G. The hallmarks of aging. *Cell* 2013; 153: 1194-1217.

22. Alder JK, Hanumanthu VS, Strong MA, DeZern AE, Stanley SE, Takemoto CM, Danilova L, Applegate CD, Bolton SG, Mohr DW, Brodsky RA, Casella JF, Greider CW, Jackson JB, Armanios M. Diagnostic utility of telomere length testing in a hospital-based setting. *Proc Natl Acad Sci U S A* 2018; 115: E2358-E2365.

23. Xu J, Woods CR, Mora AL, Joodi R, Brigham KL, Iyer S, Rojas M. Prevention of endotoxin-induced systemic response by bone marrow-derived mesenchymal stem cells in mice. *Am J Physiol Lung Cell Mol Physiol* 2007; 293: L131-141.

24. Dominici M, Le Blanc K, Mueller I, Slaper-Cortenbach I, Marini F, Krause D, Deans R, Keating A, Prockop D, Horwitz E. Minimal criteria for defining multipotent mesenchymal stromal cells. The International Society for Cellular Therapy position statement. *Cytotherapy* 2006; 8: 315-317.

25. Xu J, Gonzalez ET, Iyer SS, Mac V, Mora AL, Sutliff RL, Reed A, Brigham KL, Kelly P, Rojas M. Use of senescence-accelerated mouse model in bleomycin-induced lung injury suggests that bone marrow-derived cells can alter the outcome of lung injury in aged mice. *J Gerontol A Biol Sci Med Sci* 2009; 64: 731-739.

26. Ghosh D, McGrail DJ, Dawson MR. TGF-beta1 Pretreatment Improves the Function of Mesenchymal Stem Cells in the Wound Bed. *Front Cell Dev Biol* 2017; 5: 28.

27. Zhao L, Hantash BM. TGF-beta1 regulates differentiation of bone marrow mesenchymal stem cells. *Vitam Horm* 2011; 87: 127-141.

28. Park JS, Chu JS, Tsou AD, Diop R, Tang Z, Wang A, Li S. The effect of matrix stiffness on the differentiation of mesenchymal stem cells in response to TGF-beta. *Biomaterials* 2011; 32: 3921-3930.

29. Wiley CD, Velarde MC, Lecot P, Liu S, Sarnoski EA, Freund A, Shirakawa K, Lim HW, Davis SS, Ramanathan A, Gerencser AA, Verdin E, Campisi J. Mitochondrial Dysfunction Induces Senescence with a Distinct Secretory Phenotype. *Cell Metab* 2016; 23: 303-314.

30. Campisi J. Cellular Senescence and Lung Function during Aging. Yin and Yang. *Ann Am Thorac Soc* 2016; 13: S402-S406.

31. Wanet A, Arnould T, Najimi M, Renard P. Connecting Mitochondria, Metabolism, and Stem Cell Fate. *Stem Cells Dev* 2015; 24: 1957-1971.

32. Abe Y, Sakairi T, Beeson C, Kopp JB. TGF-beta1 stimulates mitochondrial oxidative phosphorylation and generation of reactive oxygen species in cultured mouse podocytes, mediated in part by the mTOR pathway. *Am J Physiol Renal Physiol* 2013; 305: F1477-1490.

33. Alvarez D, Levine M, Rojas M. Regenerative medicine in the treatment of idiopathic pulmonary fibrosis: current position. *Stem Cells Cloning* 2015; 8: 61-65.

34. Prockop DJ. The exciting prospects of new therapies with mesenchymal stromal cells. *Cytotherapy* 2017; 19: 1-8.

35. Prockop DJ. Inflammation, fibrosis, and modulation of the process by mesenchymal stem/stromal cells. *Matrix Biol* 2016; 51: 7-13.

36. Selman M, Pardo A. Stochastic age-related epigenetic drift in the pathogenesis of idiopathic pulmonary fibrosis. *American journal of respiratory and critical care medicine* 2014; 190: 1328-1330.

37. Bueno M, Lai YC, Romero Y, Brands J, St Croix CM, Kamga C, Corey C, Herazo-Maya JD, Sembrat J, Lee JS, Duncan SR, Rojas M, Shiva S, Chu CT, Mora AL. PINK1 deficiency impairs mitochondrial homeostasis and promotes lung fibrosis. *The Journal of clinical investigation* 2015; 125: 521-538.

38. Alder JK, Chen JJ, Lancaster L, Danoff S, Su SC, Cogan JD, Vulto I, Xie M, Qi X, Tuder RM, Phillips JA, 3rd, Lansdorp PM, Loyd JE, Armanios MY. Short telomeres are a risk factor for idiopathic pulmonary fibrosis. *Proc Natl Acad Sci U S A* 2008; 105: 13051-13056.

**FIGURE LEGENDS**

**Figure 1. B-MSCs from IPF patients show increased senescence.** (**A**) Proliferation rates of IPF B-MSC (filled circles) compared to age-matched controls (Old, open circles), showing slower proliferation rates in IPF B-MSCs compared to age-matched controls. (**B**) Representative photos of B-MSCs subjected to SA-β-galactosidase staining. (**C**) Percentage of SA-β-gal-positive cells from IPF patients and age-matched controls (Old), (mean ± SEM; n = 3). (**D**) Expression of senescence markers p21, p16^INK4A^ and p53 measured by qRT-PCR, (FC ± SEM; *p<0.05).

**Figure 2. B-MSCs from IPF patients show accumulation of DNA damage.** Immunofluorescent analysis of histone H2AX phosphorylation (γH2AX) was performed. (**A**) Representative images are shown for control (Old) and patient (IPF) cells (γH2AX, red; DAPI, blue). (**B**) Results are expressed as the percentage of the γH2AX positive cells, showing significantly more γH2AX positive IPF B-MSCs than old control cells (mean ± SEM; n = 10; **p ≤ 0.01).

**Figure 3. B-MSCs from IPF patients have a decline in their ability to differentiate.** *in vitro* adipose cell differentiation was induced in B-MSCs from age-match controls (Old) and IPF patients. Cells were incubated with adipose differentiation media for 15 days, fixed and stained with Oil Red O. Differentiation was assessed quantitatively by microscopic analysis of red pixels to measure lipid accumulation relative to the control samples (mean ± SEM; ****p ≤ 0.0001).

**Figure 4. IPF B-MSCs are impaired in migration.** Cell migration was determined by creating a wound gap and the progression of wound closure was photographed using an inverted microscope. IPF and age-matched (Old) B-MSCs were incubated in growth medium with or without 1% IPF serum (pull of n = 3) for 48 hours (bottom).

**Figure 5. Mitochondria in B-MSCs from IPF patients are more abundant but smaller compared to aged-matched controls.** (**A**) Transmission electron microscopy (TEM) (*n* = 2 and 4 per group) in B-MSC from donor control and IPF patients. Scale-bars: 500 nm. (**B**, **C**) Quantitative analysis of morphometric data from TEM images (area and length). (**D**) Mitochondrial mass determined by MitoTracker Green and normalized to cell number using DAPI (*p≤0.05, **p≤0.01).

**Figure 6. Mitochondria in B-MSCs from IPF patients have lower OCR and ECAR compared to aged-matched control.** Real time measurements (mean ± SEM, n = 4 with technical triplicates) of the mitochondrial oxygen consumption rate (OCR; pmol O_2_/min) and Extracellular Acidification Rate (ECAR; mpH/min) of B-MSC were measured under basal condition and in response to indicated mitochondrial inhibitors. OCR **(A)** and ECAR **(B)** in IPF B-MSC (continuous line) is significantly lower compared to B-MSC from age-matched controls (dashed line). The basal relationship between OCR and ECAR (**C**) of IPF B-MSCs was lower compared to control. ATP content in IPF B-MSCs **(D)** was measured by bioluminescense assay. Total ATP content was shown to be non-significantly lower in IPF B-MSCs compared to age-matched controls. To evaluate the effects of TGF-β stimulation on B-MSCs from IPF and control patients, B-MSCs were stimulated with human recombinant activated TGF-β1 (5 ng/ml). Real time measurements (mean ± SEM, n = 4 with technical triplicates) of the mitochondrial oxygen consumption rate (OCR; pmol O_2_/min) and Extracellular Acidification Rate (ECAR; mpH/min) of B-MSC were measured under basal condition and in response to indicated mitochondrial inhibitors. OCR in basal (continuous line) or TGF-β1 stimulated (dashed line) from B-MSC are shown for Control (**E, G**) and IPF (**F, H**) patients (*p<0.05, **p<0.01, ***p<0.001, ****p<0.0001).

**Figure 7. Aged B-MSCs have a decreased capacity to prevent lung fibrosis progression.** C57BL/6 mice were subjected to bleomycin injury and subsequently treated with: B-MSCs from young individuals (black), aged individuals (Old, green), IPF patients (red) or cell medium (blue) intravenously at day 0 (**A-C**) or day 7 (**D-F**). (**A**, **D**) Percent of initial body weight curves is shown. The group that received B-MSCs from young donors presented lower weight loss compared to the bleomicyn control group and mice receiving B-MSCs from other groups (old and IPF patients) have a similar weight loss. Infusion of cells at day 7 show a higher weight loss when treated with IPF-MSCs (**D**) (mean ± SEM; n = 5 in bleomycin control group and IPF B-MSC; n = 9 in young and old B-MSCs). (**B**) Masson’s trichrome staining of representative histologic sections (20x, scale bar = 100 µm). (**C**) Quantitation of hydroxyproline content in the lung (Pro-OH). (**E**) In the group that received cells at day 7, mice treated with B-MSCs from IPF donors have higher levels of expression of pro-fibrotic and pro-inflammatory genes compared to controls and young B-MSCs (*p<0.05, **p<0.01, ***p<0.001, ****p<0.0001).

**Figure 8. Stimulation of HLF with B-MSC CM from IPF patients recapitulates senescence and fibrotic phenotypes in old HLF.** Human lung fibroblasts (HLF) from a 65 yo male were stimulated with Conditioned Media (CM) from B-MSC of Old controls (73 ± 4 yo) and IPF patients (72 ± 5 yo) for two days. (**A**) HLF were subjected to SA-β-gal staining and percent of senescent cells were quantified (**B**). mRNA expression levels in stimulated HLF were determined by qRT-PCR for senescent markers (p21 and p53, **C**) and pro-fibrotic SASPs (FN1, Col1, ACTA1 and Col3, **D**) (mean ± SEM; *p≤0.05, **p≤0.01, ***p≤0.001). Treatment with non-conditioned media does not increase transcript levels in HLF (data not shown).
