# Supplementary figures and images for: Senescence of bone marrow-derived mesenchymal stem cells from patients with idiopathic pulmonary fibrosis
Source: Stem Cell Res Ther. 2018 Sep 26;9:257. doi: 10.1186/s13287-018-0970-6 (PMC6158816; doi:10.1186/s13287-018-0970-6)

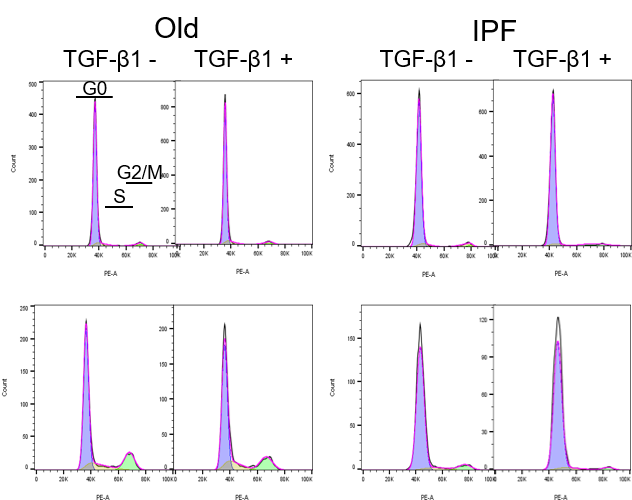

Supplement: Supplementary file 1 — Supplementary figures. (ZIP 623 kb) [file 13287_2018_970_MOESM1_ESM.zip › Suppl Figure 1_IPF MSC_NC.tif]

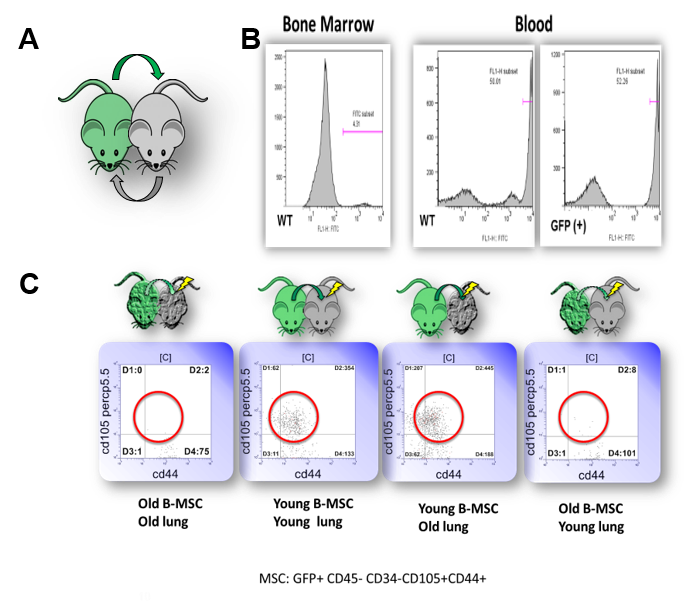

Supplement: Supplementary file 1 — Supplementary figures. (ZIP 623 kb) [file 13287_2018_970_MOESM1_ESM.zip › Suppl Figure 2_IPF MSC_NC.tif]

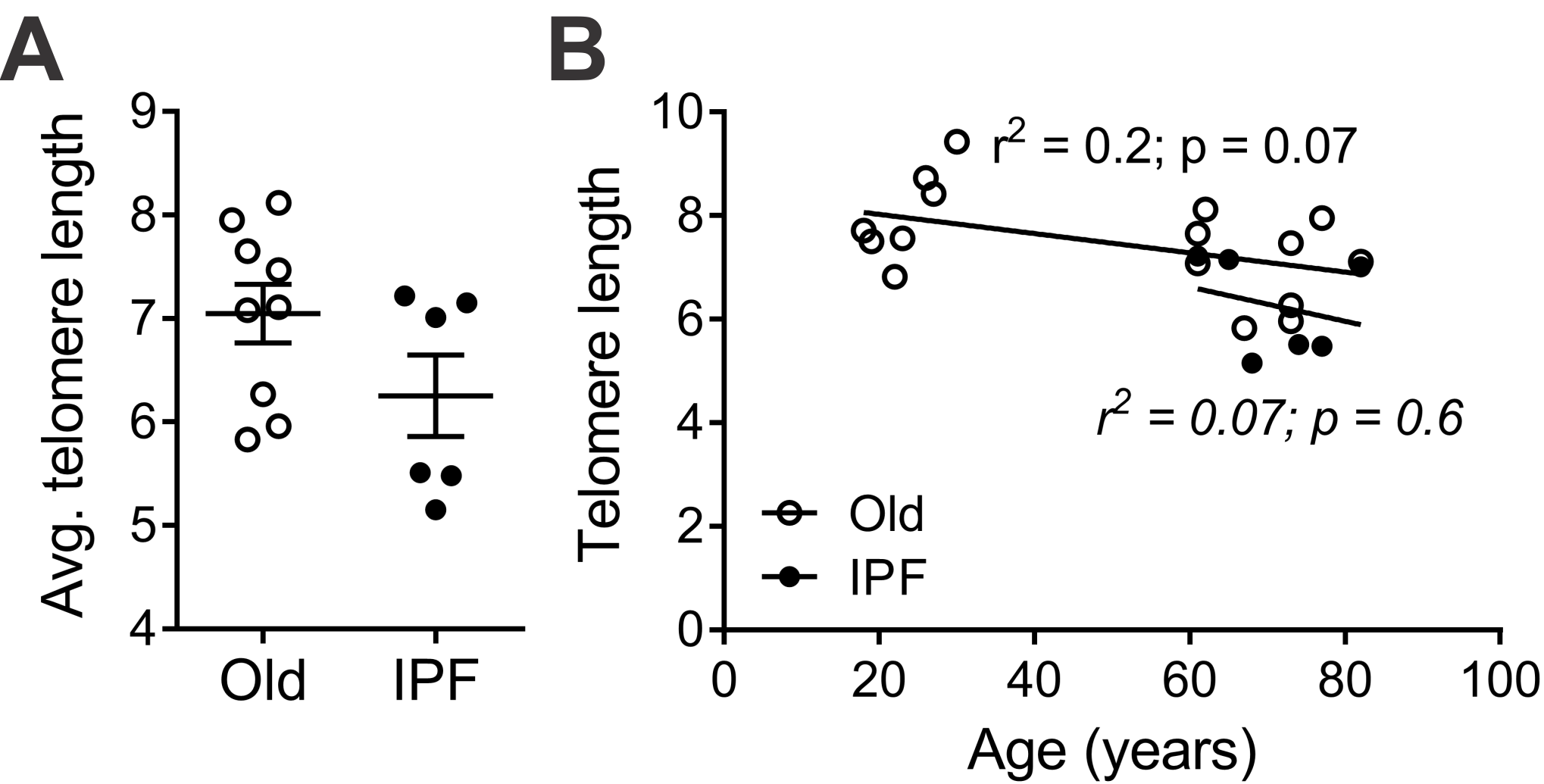

Supplement: Supplementary file 1 — Supplementary figures. (ZIP 623 kb) [file 13287_2018_970_MOESM1_ESM.zip › Supplementary Figure 3_SCR&T.tif]
